# Supplementary material for: A new extended belief rule base method based on neighborhood covering reduction for diabetes diagnosis
Source: PLoS One. 2026 May 5;21(5):e0347303. doi: 10.1371/journal.pone.0347303 (PMC13143082; doi:10.1371/journal.pone.0347303)
Supplement: S1 File — This ZIP file contains all experimental datasets used in this study, organized as follows: 1. Original Data: Sourced from the Medical City Hospital/Al-Kindy Teaching Hospital (Mendeley Data) and the UCI Machine Learning Repository. 2. Section 4.1 Datasets: Training and testing splits for diabetes diagnosis with test set proportions of 0.2, 0.25, 0.3, 0.35, and 0.4 (e.g., Dataset of Diabetes_train_0.2.xlsx). 3. Section 4.2 Datasets: Validation datasets including Iris, Banknote, Ecoli, and Newthyroid with a 0.3 test split. All processed datasets are restricted to two premise attributes to ensure a fair comparison by eliminating the influence of feature selection, thereby highlighting the performance of the NCR-EBRB model. (ZIP) [file pone.0347303.s001.zip › README.docx]

This study employed two datasets.
The first was collected by the laboratory of Medical City Hospital and the Specialized Center for Endocrinology and Diabetes at Al-Kindy Teaching Hospital, and is publicly available in the Mendeley Data repository (<https://data.mendeley.com/datasets/wj9rwkp9c2/1>).
The second dataset was obtained from the UCI Machine Learning Repository (<https://archive.ics.uci.edu/>) for experimental validation.

All original data are stored in the **origin** folder.

For the experiments in Section 4.1, the training and testing sets are provided in the current directory with the following filenames:

- Dataset of Diabetes_train_0.2.xlsx / Dataset of Diabetes_test_0.2.xlsx
- Dataset of Diabetes_train_0.25.xlsx / Dataset of Diabetes_test_0.25.xlsx
- Dataset of Diabetes_train_0.3.xlsx / Dataset of Diabetes_test_0.3.xlsx
- Dataset of Diabetes_train_0.35.xlsx / Dataset of Diabetes_test_0.35.xlsx
- Dataset of Diabetes_train_0.4.xlsx / Dataset of Diabetes_test_0.4.xlsx

Here, the suffix 0.x denotes the proportion of samples allocated to the test set (e.g., 0.2 = 20%), while _train and _test indicate the training and test splits, respectively.

For the experiments in Section 4.2, the following datasets are included in the current directory:

- Iris_train_0.3.xlsx / Iris_test_0.3.xlsx
- Banknote_train_0.3.xlsx / Banknote_test_0.3.xlsx
- Ecoli_train_0.3.xlsx / Ecoli_test_0.3.xlsx
- Newthyroid_train_0.3.xlsx / Newthyroid_test_0.3.xlsx

The naming convention follows the same pattern: the prefix indicates the dataset name, 0.3 specifies a 30% test split, and the suffix identifies the data split type.

**It should be noted that, unlike the original datasets, all datasets used in our experiments contain only two premise attributes. This design eliminates the influence of feature selection, thereby ensuring a fair comparison and better highlighting the superiority of the proposed NCR-EBRB model.**
