# Supplementary material for: A new extended belief rule base method based on neighborhood covering reduction for diabetes diagnosis
Source: PLoS One. 2026 May 5;21(5):e0347303. doi: 10.1371/journal.pone.0347303 (PMC13143082; doi:10.1371/journal.pone.0347303)
Supplement: Attachment — Submitted filename: Response_to_Reviewers_auresp_2.docx [file pone.0347303.s004.docx]

**^[1]^Reviewer #1: The following points should be addressed to improve the clarity, rigor, and overall impact of the manuscript:**

**1.Data Description Inaccuracy (Section 4.1):**

Comment: The text states, "The dataset comprises 1000 patients... with 844, 103, and 5 patients, respectively." This sums to 952, not 1000. Please verify and correct the total number of samples in the dataset to ensure accuracy.

**The author's answer:**
We sincerely thank the reviewer for identifying the numerical inconsistency. The original statement—"…with 844, 103, and 5 patients, respectively"—contained a typographical error in the count for the "predicted diabetic" class. This has been corrected to "53" in the revised manuscript:

"The dataset comprises 1000 patients and covers three classes, namely, diabetic, nondiabetic, and predicted diabetic, with 844, 103, and 53 patients, respectively. Detailed information on the data parameters is presented in Table 1. "

The updated numbers now correctly sum to 1000 and align with the data reported in Table 1.

**2.Formula Numbering:**

Comment: There is a duplication in equation numbering; two distinct equations are both labeled as "(7)". Please renumber all equations sequentially throughout the manuscript to avoid confusion.

**The author's answer:**

We appreciate the reviewer’s careful inspection. The duplicated equation number has been corrected, and all equations are now numbered sequentially throughout the manuscript.

**3.Terminology Consistency:**

Comment: The model name alternates between the full form "Extended Belief Rule Base" and the acronym "EBRB". For consistency and readability, it is recommended to use the acronym "EBRB" throughout the manuscript after it is first defined in the abstract or introduction.

**The author's answer:**

Thank you for this helpful comment. We have revised the manuscript to consistently use the acronym “EBRB” after its initial definition.

**4.Lack of Optimization Details (P-CMA-ES):**

Comment: The description of the P-CMA-ES optimization process lacks critical implementation details, such as the termination criteria and population size. To enhance reproducibility, please specify all key hyperparameters used. Furthermore, providing a convergence curve of the optimization algorithm would help demonstrate that a stable solution was reached.

**The author's answer:**

We appreciate the reviewer’s insightful comment. To improve reproducibility, we have revised the manuscript to explicitly state the key hyperparameters and termination condition of the P-CMA-ES algorithm used to train the NCR-EBRB model.

Specifically:

1) Population size:  , where  is the number of decision variables;
2) Initial step size:;
3) Termination criterion: The optimization stops after a fixed budget of  function evaluations (no early stopping was applied).

We have decided not to include the optimization convergence curve in the revised manuscript, as the training loss alone is insufficient to determine optimal iteration counts or solution quality in classification tasks. Specifically, we avoid basing iteration termination on loss minimization alone, since early stopping solely at the point of minimal training loss may inadvertently lead to overfitting. Instead, our experimental protocol employs a multi-metric evaluation framework—incorporating classification accuracy, F1-score, and generalization performance on validation sets—to guide model selection and iteration termination. This approach ensures that our stopping criteria align with practical performance rather than purely minimizing the loss function. We believe this clarification better reflects our methodology and prevents potential misinterpretation of the optimization process.

These details have been added to Section 4.1.2 of the revised manuscript, which now reads as follows:

The parameters of the NCR-EBRB model established in Step 5 are optimized using the P-CMA-ES. The algorithm employs a population size of  (where  is the number of decision variables), an initial step size of  , and runs for a maximum of  function evaluations as the termination criterion. The resulting optimized NCR-EBRB model is then used for inference.

**5.Need for Ablation Study:**

Comment: The individual contributions of the NCR module and the P-CMA-ES optimization to the model's final performance are not evaluated. To disentangle their effects, it is strongly recommended to incorporate an ablation study. This should compare the performance of:
The pure EBRB model (without rule reduction).
The NCR-EBRB model (with rule reduction but without parameter optimization).
The full NCR-EBRB model (with both rule reduction and optimization).
This analysis is crucial for validating the necessity of each proposed component.

**The author's answer:**

We sincerely thank the reviewer for this critical suggestion. We have added a comprehensive ablation study (Section 4.1.5) that:

1) Effectiveness of the NCR Module: The NCR module alone consistently improves model accuracy compared to the Pure EBRB. For instance, at the 25% test split, the NCR-EBRB (without optimization) achieved an accuracy of 0.9398, which is 2.01 percentage points higher than the 0.9197 of the Pure EBRB. This reflects the module's ability to prune redundant rules while effectively preserving decision fidelity.
2) Necessity of P-CMA-ES Optimization: Incorporating P-CMA-ES further unlocks the model's performance potential. At the same 25% split, the full NCR-EBRB increased the accuracy from 0.9398 to 0.9759. This significant gain of 3.61 percentage points demonstrates the necessity of fine-grained parameter adaptation for achieving optimal predictive performance.
3) Overall Synergy: The synergy between rule reduction and parameter optimization is evident across all data splits. Notably, the full NCR-EBRB model achieved its peak performance at the 25% split (0.9759), outperforming the Pure EBRB by 5.62 percentage points.

These results explicitly validate that both proposed components are indispensable for the high accuracy and interpretability of the framework.

4.1.4 Ablation Study

To rigorously validate the individual contributions of the NCR module and P-CMA-ES optimization, a comprehensive ablation study was conducted across varying test set proportions (20%–40%), comparing three configurations: (1) Pure EBRB (no rule reduction/optimization), (2) NCR-EBRB without optimization (rule reduction only), and (3) the full NCR-EBRB (with both components).

As shown in Table 4, the NCR module alone improved the accuracy over the Pure EBRB at the 20% test split (0.9394 vs. 0.9343), reflecting effective rule pruning while preserving decision fidelity. Incorporating P-CMA-ES further enhanced performance significantly, increasing the accuracy to 0.9747 compared to 0.9394 for the unoptimized NCR-EBRB at the same split, indicating its necessity for fine-grained parameter adaptation.

Notably, the full NCR-EBRB model achieved its peak performance at the 25% split with an accuracy of 0.9759, outperforming the Pure EBRB (0.9197) by 5.62 percentage points. This confirms that both modules are indispensable for optimal predictive performance. Overall, the ablation results verify that NCR provides efficient structural reduction while P-CMA-ES unlocks additional optimization potential, and their combination yields robust performance across different partitioning ratios.

**Table 4: Ablation Study of NCR-EBRB under Varying Test Set Partitioning Ratios**

| Percentage | Model | Accuracy | Macro-Precision | Macro-Recall |
| --- | --- | --- | --- | --- |
| 0.2 | NCR-EBRB | **0.9747** | **0.9229** | **0.9607** |
|  | NCR-EBRB (without optimization) | 0.9394 | 0.8637 | 0.8087 |
|  | Pure EBRB | 0.9343 | 0.7810 | 0.8968 |
|  |  |  |  |  |
| 0.25 | NCR-EBRB | **0.9759** | **0.9268** | **0.9665** |
|  | NCR-EBRB (without optimization) | 0.9398 | 0.8454 | 0.8817 |
|  | Pure EBRB | 0.9197 | 0.7411 | 0.8240 |
|  |  |  |  |  |
| 0.3 | NCR-EBRB | **0.9732** | **0.9555** | **0.9058** |
|  | NCR-EBRB (without optimization) | 0.9329 | 0.8104 | 0.7895 |
|  | Pure EBRB | 0.9329 | 0.7675 | 0.8273 |
|  |  |  |  |  |
| 0.35 | NCR-EBRB | **0.9628** | **0.8825** | **0.9169** |
|  | NCR-EBRB (without optimization) | 0.9284 | 0.8153 | 0.7502 |
|  | Pure EBRB | 0.9255 | 0.7489 | 0.7712 |
|  |  |  |  |  |
| 0.40 | NCR-EBRB | 0.9549 | 0.9180 | 0.8733 |
|  | NCR-EBRB (without optimization) | 0.9348 | 0.8290 | 0.8064 |
|  | Pure EBRB | 0.9248 | 0.7588 | 0.8072 |

**6.Missing Limitations Analysis:**

Comment: The conclusion section would be strengthened by a discussion of the model's limitations. For instance, the performance appears sensitive to hyperparameters (e.g., the rule fusion threshold t). Furthermore, as hinted at in Table 3 with the 40% test set, rule reduction may lead to performance degradation when training data is scarce. Acknowledging these limitations provides a more balanced and scientifically honest perspective.

**The author's answer:**

We fully agree with the reviewer that a transparent discussion of limitations enhances scientific rigor. Accordingly, we have added a dedicated paragraph in the Conclusion section (now the second paragraph of the revised conclusion) that explicitly addresses both points raised:

1. Sensitivity to hyperparameter λ: We acknowledge that the rule fusion threshold λ directly controls the size of the final rule base and that an inappropriate choice can negatively impact accuracy—either by discarding informative rules or retaining redundant ones. This is now clearly stated, and we further note that this limitation is mitigated in our experiments through a systematic hyperparameter tuning strategy that consistently identifies near-optimal λ values across all datasets (as detailed in Section 4.2.3).
2. Performance under data scarcity: While the reviewer correctly observes a potential risk of performance degradation due to aggressive rule reduction when training data are limited, we clarify that this scenario was intentionally explored in Table 3 (40% test set → 60% training data). Our results show a modest accuracy drop, confirming that extreme data scarcity can challenge the stability of rule reduction. However, under standard data splits (e.g.80% training), the model remains robust. We have now explicitly referenced this trade-off in the revised conclusion to provide a more nuanced and honest assessment of the method’s applicability boundaries.

These additions ensure that readers understand both the strengths and contextual constraints of NCR-EBRB.

**7.Strengthening the Conclusion:**

Comment: The conclusion should more directly address the research objectives stated in the introduction. If "improving interpretability" was a key goal, the discussion should explicitly state how NCR-EBRB achieves this. For example, quantify the improvement by stating that the model "reduced the number of rules by 50% while maintaining 95% accuracy," thereby enhancing transparency and traceability.

**The author's answer:**

Thank you for this valuable suggestion. We have significantly strengthened the conclusion to directly connect the method’s design to the core objective of enhancing interpretability, using actual experimental results from our study (not illustrative examples).

Specifically, we now explicitly state in the third paragraph of the revised conclusion:

The core innovation of NCR-EBRB—rule base simplification—significantly enhances interpretability without sacrificing performance. By reducing the rule count from 702 to 125 (an 82.2% reduction), the model achieves a highly transparent and clinically actionable rule set while maintaining high accuracy. This drastic simplification directly fulfills the primary objective of improving model interpretability, enabling medical experts to trace and validate diagnostic reasoning with confidence.

Thus, the revised conclusion now clearly demonstrates *how* and *to what extent* NCR-EBRB improves interpretability, grounded in empirical evidence rather than hypothetical claims.

**Reviewer #2:**

**1. Formal theoretical justification for integrating NCR with EBRB is lacking**

The manuscript introduces Neighborhood Covering Reduction (NCR) for EBRB rule reduction, yet the presentation remains largely algorithmic. A theoretical analysis of how this reduction affects the reasoning consistency or completeness of the EBRB system is absent. It is recommended that the authors include a discussion on topics such as rule coverage sufficiency, information preservation, or potential error bounds to strengthen the theoretical foundation.

**The author's answer:**

We sincerely thank the reviewer for highlighting this critical gap. The theoretical foundation of NCR-EBRB integration has been strengthened in Section 3.2.1 (newly added paragraph at the end), which explicitly addresses:

NCR ensures reasoning completeness by maintaining rule coverage sufficiency (i.e., all samples are covered by at least one rule).

This revision provides the formal theoretical grounding requested, directly linking NCR’s algorithmic design to EBRB’s reasoning robustness.

**2. Semantic consistency after rule reduction is not clearly addressed**

In EBRB systems, rules encapsulate expert semantic knowledge, while NCR operates as a distance based rule selection method. The manuscript does not examine whether rule removal via NCR preserves the original semantic integrity of the expert knowledge. A discussion on the semantic preservation characteristics of the proposed NCR EBRB framework would therefore be valuable.

**The author's answer:**
We thank the reviewer for emphasizing this critical aspect. Section 3.2.1 (newly added paragraph) now explicitly clarifies that NCR:

1) Preserves rule structure (including semantic attributes and belief distributions) without modification;
2) Prioritizes expert-defined decision boundaries via classification margin analysis (Eq. 13);

Thus, rule removal via NCR does not compromise the original semantic knowledge embedded in EBRB rules.

**3. No automatic selection mechanism for the rule fusion threshold λ**

Although Section 4.2 analyzes the influence of the rule fusion threshold λ, its determination in practice still depends on manual tuning. It is suggested that the authors consider validation based or adaptive strategies to automatically select an appropriate λ, thereby improving the usability and robustness of the method.

**The author's answer:**

We sincerely thank the reviewer for raising this important point. We fully agree that an automatic mechanism for selecting λ would enhance the practical usability of our method. In the current manuscript, we intentionally focused on establishing the theoretical foundation and semantic validity of the NCR-EBRB framework (Sections 3.2.1, 4.3), and λ’s manual tuning was based on the empirical validation shown in Fig. 7 (λ∈ [0.3, 0.7] consistently yields robust results across datasets).

We acknowledge that this manual tuning is a limitation, and we are actively developing an adaptive strategy for λ selection using meta-learning and cross-validation. This work will be detailed in our upcoming paper, where we aim to provide a data-driven solution for λ determination.

**4. Worst case computational complexity of the rule reduction algorithm is not analyzed**

Algorithm 1 employs a greedy forward search strategy; however, its worst case time and space complexity for large scale rule bases is not discussed. Providing a formal complexity analysis would help clarify the scalability of the proposed approach.

**The author's answer:**
We sincerely thank the reviewer for raising this important point. We fully agree that worst-case complexity analysis is essential. The algorithm achieves time complexity (Step 6), as explained at the end of the first paragraph of Section 3.2.2.

**5. Identical Precision and Recall values require explanation**

In Tables 3 and 5, Precision and Recall are repeatedly reported as equal. Such a result is uncommon in general classification tasks and warrants clarification—for instance, whether it stems from macro averaging, class imbalance, or specific implementation details.

**The author's answer:**
**Author’s Response:** We thank the reviewer for this insightful comment. We have thoroughly addressed the concerns regarding the evaluation metrics as follows:

1. **Implementation of Macro-averaging:** To ensure the technical rigor and clinical relevance of our diagnostic model, we have updated our evaluation methodology from micro-averaging to **macro-averaging** in the revised manuscript. Unlike micro-averaging, which can be dominated by the majority class, macro-averaging treats each diagnostic category (e.g., healthy vs. diabetic) with equal weight. This provides a more balanced and transparent assessment of the model’s performance across all patient groups.
2. **Clarification of Metric Disparity:** Following the adoption of the macro-average scheme, the **Precision** and **Recall** values in **Tables 3, 4, and 6** are no longer identical. Each metric now independently reflects a specific dimension of the model's performance: Precision represents the exactness (clinical positive predictive value), while Recall represents the completeness (sensitivity).
3. **Analysis of Updated Results:** In the revised results, the Overall Accuracy is generally higher than the Macro-averaged Precision and Recall. This is a standard and expected phenomenon in imbalanced medical datasets, where the majority class influences overall accuracy more significantly. By reporting macro-averaged metrics, we provide a more stringent and honest evaluation of the NCR-EBRB method’s robustness.
4. **Table Revisions:** All experimental data in **Tables 3, 4, and 6** have been recalculated using the updated macro-averaging logic and are accurately reflected in the revised manuscript.

**6. Parameter settings of comparative models are not disclosed**

The parameter configurations for the comparative models (SVM, KNN, Bagging Tree, and Boosting Tree) are not provided. This omission may compromise the fairness and reproducibility of the experimental comparisons. Reporting these details is essential for rigorous evaluation.

**The author's answer:**
We thank the reviewer for emphasizing the importance of reproducibility in comparative model evaluation. In our experiments, the baseline classifiers were implemented with fixed configurations and without hyper-parameter tuning. We apologize for not explicitly describing these configurations in the manuscript. The details have now been included in Section X to ensure both transparency and reproducibility. Specifically:

SVM was implemented under MATLAB’s default multi-class scheme using linear base classifiers.

The Decision Tree baseline adopted a moderate tree complexity with controlled depth and minimum leaf size.

KNN employed a neighborhood size of five.

The Bagging Tree ensemble consisted of twenty decision trees.

The Boosting Tree model utilized decision trees as weak learners with moderate complexity.

These configurations are consistent with commonly used baseline settings in the literature and were applied uniformly across comparative experiments. The manuscript has been updated accordingly in Section 4.1.3

**7. Section lengths are imbalanced**

Section 3.2 is relatively lengthy compared to the concise Conclusion section. Rebalancing the manuscript structure would enhance overall readability and coherence.

**The author's answer:**
We thank the reviewer for the suggestion. The conclusion section has been expanded into four paragraphs to provide a more comprehensive and balanced discussion. which now reads as follows:

5.Conclusion

This study proposes a feature selection-based neighborhood coverage reduction EBRB (NCR-EBRB) method, which simultaneously addresses critical challenges in feature definition and initial parameter configuration. Experimental validation across four benchmark datasets demonstrates its robust performance and strong application potential in classification tasks.

The model's performance is inherently sensitive to the hyperparameter $\text{λ}$ (rule fusion threshold), as $\text{λ}$ directly governs the final rule base size (Section 4.2.3). An ill-chosen $\text{λ}$ may discard critical diagnostic rules or retain redundant ones, potentially degrading accuracy.

The core innovation of NCR-EBRB—rule base simplification—significantly enhances interpretability without sacrificing performance. By reducing the rule count from 702 to 125 (an 82.2% reduction), the model achieves a highly transparent and clinically actionable rule set while maintaining high accuracy. This drastic simplification directly fulfills the primary objective of improving model interpretability, enabling medical experts to trace and validate diagnostic reasoning with confidence.

To further strengthen stability, future work will develop intelligent parameter adjustment mechanisms to reduce manual tuning reliance. We also plan to integrate local interpretability techniques,^[35]^ for deeper analysis of the NCR-EBRB decision process, enhancing its clinical utility and transparency.

8. Inconsistent figure/table numbering and textual references

Some figures and tables are inconsistently numbered or referenced in the main text. A thorough check to ensure consistency is recommended.

**The author's answer:**

We thank the reviewer for the careful observation. The inconsistent figure and table numbering has been corrected throughout the manuscript.

**Reviewer #3: This manuscript proposes an extended belief rule base (EBRB) framework for diabetes diagnosis. The topic is relevant to clinical decision support, and the integration of rule-base reduction with an interpretable EBRB-style model is potentially useful. The paper is suitable for publication after minor revisions that address the following issues.**

1. The manuscript states that NCR-EBRB outperforms other benchmark models across the four UCI datasets. However, Table 5 indicates that for Banknote, KNN (0.9075) exceeds NCR-EBRB (0.8929).

**The author's answer:**
We thank the reviewer for pointing out this discrepancy. After rechecking the experiments, we found that the results for KNN and NCR-EBRB were inadvertently reversed in Table 5. We sincerely apologize for the oversight. The corrected results are now included in the revised manuscript.

2. Equation (7) is used for the XGBoost gain function and later reused for the EBRB rule expression.

**The author's answer:**
Thank you for your observation. The duplicated equation number has been corrected.

3. Algorithm 1 repeats step number “7” and has minor formatting issues.

**The author's answer:**
We thank the reviewer for pointing out the duplicated step numbering and formatting issues in Algorithm 1. We have thoroughly revised the algorithm to ensure consistent numbering, improved layout, and clearer logical structure. In particular, the two repeated “Step 7” entries have been corrected, which now reads as follows:

Algorithm 1: EBRB rule reduction algorithm based on neighborhood covering

Input: Original rule base

Output: rule set

1: compute the margins of rules,if,set

2: compute of sample

3:,

4: compute the number of the samples covered by each covering element in

5: While

6: select the covering element covering the largest number of samples

7: add a rule into

8: remove if

9: end

4. Several visible typographical and spacing issues appear.

**The author's answer:**

We thank the reviewer for the careful proofreading. All visible typographical and spacing issues have been corrected in the revision.
